# Spatiotemporal regulation of ventilator lung injury resolution by TGF-β1+ regulatory B cells via macrophage vesicle-nanotherapeutics

**DOI:** 10.3389/fimmu.2025.1635178

**Published:** 2025-07-10

**Authors:** Ren Jing, Xiaoting Liao, Jianlan Mo, Sheng He, Xianlong Xie, Zhaokun Hu, Linghui Pan

**Affiliations:** 1 Guangxi Clinical Research Center for Anesthesiology, Guangxi Medical University Cancer Hospital, Nanning, China; 2 Department of Breast and Thyroid Surgery, South China Hospital, Medical School, Shenzhen University, Shenzhen, China; 3 Department of Anesthesiology, Guangxi Medical University Cancer Hospital, Nanning, China; 4 Guangxi Engineering Research Center for Tissue & Organ Injury and Repair Medicine, , Guangxi Medical University Cancer Hospital, Nanning, China; 5 Guangxi Key Laboratory for Basic Science and Prevention of Perioperative Organ Disfunction, Guangxi Medical University Cancer Hospital, Nanning, China; 6 Department of Anesthesiology, Guangxi Maternal and Child Health Hospital, Nanning, China; 7 The First Affiliated Hospital, Department of Anesthesiology, Hengyang Medical School, University of South China, Hengyang, Hunan, China; 8 Department of Intensive Care Unit, Guangxi Medical University Cancer Hospital, Nanning, China

**Keywords:** ventilation-induced lung injury, transforming growth factor-β1, regulatory B cells, immunoresolution, nanoparticles

## Abstract

**Background:**

Regulatory B cells (Breg) critically orchestrate inflammatory resolution and tissue repair. This study investigates the therapeutic potential of transforming growth factor (TGF)-β1-producing Bregs in ventilator-induced lung injury (VILI), leveraging biomimetic nanotechnology to overcome limitations of conventional cytokine delivery.

**Methods:**

We engineered macrophage-derived microvesicle-encapsulated nanoparticles (TMNP) for pH-responsive, spatiotemporally controlled TGF-β1 release. Therapeutic efficacy was evaluated in a murine VILI model through longitudinal immunophenotyping, histopathology, and cytokine profiling at post-ventilation days 1 and 10 (PV1d, PV10d).

**Results:**

VILI triggered biphasic pulmonary Breg expansion (PV1d: 7.83-fold *vs*. controls, *P* < 0.001; PV10d resurgence) coinciding with peak injury. TMNP administration induced sustained TGF-β1 bioavailability (PV10d: 3.6-fold *vs*. free cytokine, *P* < 0.001), attenuating histopathology (22.5% reduction in alveolar hemorrhage, *P* < 0.01) and suppressing IL-6/TNF-α (*P* < 0.01). Treatment concomitantly expanded Breg populations and modulated T cell subset.

**Conclusion:**

TMNP orchestrates Breg-mediated immunoresolution through precision cytokine delivery and lymphocyte modulation, enabling dual-phase protection against ventilation-associated immunopathology. This paradigm represents a transformative approach for acute respiratory distress management.

## Graphical Abstract

**Figure:**
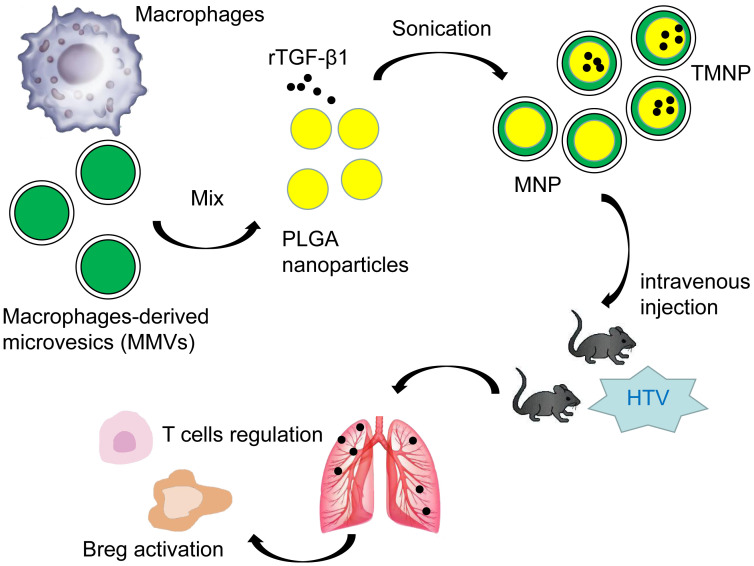

## 1 Introduction

Acute lung injury (ALI) and its severe manifestation, acute respiratory distress syndrome (ARDS), constitute life-threatening conditions exacerbated by global health crises like COVID-19 (1, 2). Mechanical ventilation, while essential for ARDS management, paradoxically induces ventilator-induced lung injury (VILI) through synergistic biomechanical forces and inflammatory cascades that disrupt alveolar-capillary integrity (3, 4). Despite lung-protective ventilation strategies, the immunological mechanisms governing injury resolution remain poorly defined, impeding targeted therapeutic development.

Regulatory B cells (Bregs) represent a pivotal immunomodulatory axis that coordinates inflammatory resolution via cytokine secretion (e.g., transforming growth factor [TGF]-β1) and lymphocyte modulation (5, 6). Although Bregs attenuate inflammation in autoimmune and infectious contexts (7–9), their spatiotemporal dynamics and TGF-β1-mediated functions in VILI remain uncharacterized. This knowledge gap persists despite TGF-β1’s documented role in mitigating ALI and directing macrophage polarization toward reparative phenotypes (10–12). Crucially, TGF-β1’s therapeutic potential is limited by its transient bioavailability (t1/2 ≈ 2 min *in vivo*) (13, 14), necessitating innovative delivery platforms.

To address these critical limitations, we engineered macrophage-derived microvesicles (MMVs)-camouflaged nanoparticles (TMNP) encapsulating TGF-β1-loaded Carboxy-terminated poly(lactic-co-glycolic acid) (PLGA) cores—a biomimetic platform leveraging MMVs’ inherent macrophage tropism for targeted alveolar delivery, pH-responsive release kinetics to overcome cytokine instability, and synergistic immunomodulatory properties. Our study specifically interrogates the unexplored role of TGF-β1^+^Bregs in VILI pathogenesis, TMNP’s capacity to sustain TGF-β1 bioavailability, and mechanisms underlying Breg-mediated immunoresolution.

## 2 Materials and methods

### 2.1 Reagents and chemicals

PLGA (50:50 lactide:glycolide ratio, MW 38-54 kDa, LACTEL B6013-2) served as the polymer matrix. Recombinant mouse TGF-β1 (BioLegend 763102), cytochalasin B (Abcam ab143482), and uranyl acetate (Sigma-Aldrich 73943) were utilized. All solvents including chloroform (HPLC grade, Sigma-Aldrich 650498) and dimethyl sulfoxide (DMSO, ThermoFisher D12345) met analytical standards.

### 2.2 Animal subjects

Male C57BL/6 mice (4–6 weeks, 25 ± 5 g) from Guangxi Medical University’s Animal Center (Nanning, China) were maintained under specific pathogen-free conditions. All procedures complied with China’s Laboratory Animal Welfare Guidelines under IACUC protocolKY-2022-288.

### 2.3 MMVs isolation

RAW 264.7 macrophages (Cell Bank of Chinese Academy of Sciences) were cultured in advanced Dulbecco’s Modified Eagle Medium (DMEM; Gibco, 12491015) supplemented with 10% fetal bovine serum (Gibco, 10270106) and 1% penicillin/streptomycin (Gibco, 15140122). MMVs were generated via cytochalasin B-induced membrane blebbing (15): cells were treated with 10 μg/ml cytochalasin B in serum-free DMEM for 1 h at 37°C. Following membrane detachment, suspensions underwent sequential centrifugation (5,000 ×g, 10 min; 17,000 ×g, 15 min) with ethylenediaminetetraacetic acid -containing MilliQ washes. Microvesicle protein content was quantified via BCA assay (ThermoFisher, 23227) and validated through CD9/CD63 immunoblotting.

### 2.4 Nanoparticle synthesis

#### 2.4.1 PLGA core fabrication

Carboxy-terminated PLGA dissolved in chloroform (20 mg/ml) was emulsified with 2.5 μg recombinant mouse TGF-β1using a water-in-oil-in-water double emulsion technique (15). Primary emulsions were sonicated (BILON-1000Y probe sonicator, 60% amplitude, 10-s pulses on ice bath) and introduced into 2% polyvinyl alcohol solution. After 3 h solvent evaporation under mechanical stirring (500 rpm), nanoparticles were collected by centrifugation (15,000 ×g, 30 min, 4°C) and washed thrice with MilliQ water.

#### 2.4.2 MMVs coating

Lyophilized PLGA nanoparticles were combined with MMVs at 1:10 w/w protein:PLGA ratio. Sonication (GuTel GT-100 water bath, 40 kHz, 3 min) generated TGF-β1-loaded MMV- nanoparticles (TMNP), which underwent lyophilization (Christ Alpha 2-4 LSCplus, -50°C, 0.05 mBar, and 48 h). MMV- nanoparticles without TGF-β1 loading (MNP) was defined as control.

### 2.5 Nanoparticle characterization

Morphological analysis employed transmission electron microscopy (Hitachi HT7800) with uranyl acetate negative staining. Hydrodynamic diameter and zeta potential were determined via dynamic light scattering (Malvern Zetasizer) and nanoparticle tracking analysis (ZetaView^®^), respectively. Encapsulation efficiency (89.7% ± 2.4%) was calculated as (encapsulated TGF-β1/total TGF-β1) × 100 after DMSO dissolution, while drug-loading capacity (4.31% ± 0.18%) represented (encapsulated TGF-β1/TMNP mass) × 100, both quantified by enzyme linked immunosorbent assay (ELISA).Stability assessments monitored hydrodynamic diameter at 4°C over 7 days and turbidity at 560 nm. Release kinetics in 0.5% Tween-80/PBS (pH7.4) at 37°C demonstrated sustained release >96 h via ELISA quantification.

### 2.6 VILI protocol

Anesthetized mice (tribromoethanol 20 mg/kg i.p.) underwent orotracheal intubation and mechanical ventilation (SAR-100) under high tidal volume (HTV: 20 mL/kg) or normal tidal volume (NTV: 7 mL/kg) for 4 h. Cohorts (*n* = 4/group) were euthanized at post-ventilation day 1 (PV1d) and day 10 (PV10d). Sham controls received intubation without ventilation. Bronchoalveolar lavage fluid (BALF) from left lungs, serum, and lung tissue were stored at -80 ˚C; right upper/middle lobes underwent frozen sectioning and TEM processing.

### 2.7 Therapeutic administration

TMNP (0.5 mg/kg), MNP (0.5 mg/kg), or free recombinant TGF-β1 (40 μg/kg) (16, 17) in 50 μL saline were administered intravenously pre-ventilation. Vehicle controls received saline alone.

### 2.8 Pathological assessments

#### 2.8.1 Edema quantification

Lung wet/dry weight ratios were calculated after 48 h desiccation at 60°C.

#### 2.8.2 Inflammation profiling

BALF protein (BCA assay), cellular composition (automated cytometry), and cytokine levels (IL-1β, IL-6, TNF-α, TGF-β1; ELISA) were analyzed.

#### 2.8.3 Histopathological evaluation

H&E-stained sections were scored for alveolar hemorrhage, neutrophil infiltration, and hyaline membrane using established criteria (10, 11). Ultrastructural analysis employed TEM (Hitachi HT7800).

### 2.9 Immunophenotyping

Lung/spleen single-cell suspensions were prepared via enzymatic digestion (0.1 mg/ml Dispase II, 2000 U/ml DNase I, 0.2% collagenase). After Fc receptor blocked (TruStain FcX™ PLUS), cells were stained with fluorochrome-conjugated antibodies (Biolegend/BD Biosciences: CD5-PE, CD19-APC, CD4-FITC, CD8a-APC, CD44-PE/Cy7, LAP-PE) and analyzed by flow cytometry (CytoFLEX LX, Beckman Coulter).

### 2.10 Multiplex immunofluorescence

Frozen sections underwent fixation (4% paraformaldehyde), permeabilization (0.2% Triton X-100), and blocking (3% BSA/3% goat serum). Sequential incubations with primary antibodies (anti-CD19, LAP, CD44) and Alexa Fluor-conjugated secondaries preceded DAPI nuclear counterstaining. Imaging utilized a Zeiss LSM980 Airyscan confocal microscope.

### 2.11 Statistical analysis

Bioinformatic analyses employed DAVID (v6.8) for pathway enrichment and STRING (v11.5) for protein interactions (combined score >0.4). Following normality assessment (Shapiro-Wilk), two-tailed t-test (two groups), one-way ANOVA with Tukey’s *post-hoc* (multi-group), or two-way ANOVA with Bonferroni correction (time courses) were applied. Data represent mean ± SEM; *P*<0.05 defined statistical significance.

## 3 Results

### 3.1 Spatiotemporal dynamics of CD19^high^CD44^high^TGF-β1+ Breg in VILI resolution

High-throughput immunophenotyping revealed selective upregulation of tissue migration receptors (CD44, CX3CR1) (18, 19) on splenic Bregs following 4-hour HTV ventilation, while canonical B cell markers remained unchanged (**Supplementary Figure S1A**). Protein interaction networks demonstrated direct associations between CD19, CD44, and TGF-β1 (**Supplementary Figure S1B**), with pathway enrichment implicating B cell receptor signaling and epithelial-mesenchymal transition regulation (**Supplementary Figures S1C, D**).

**Figure 1:**
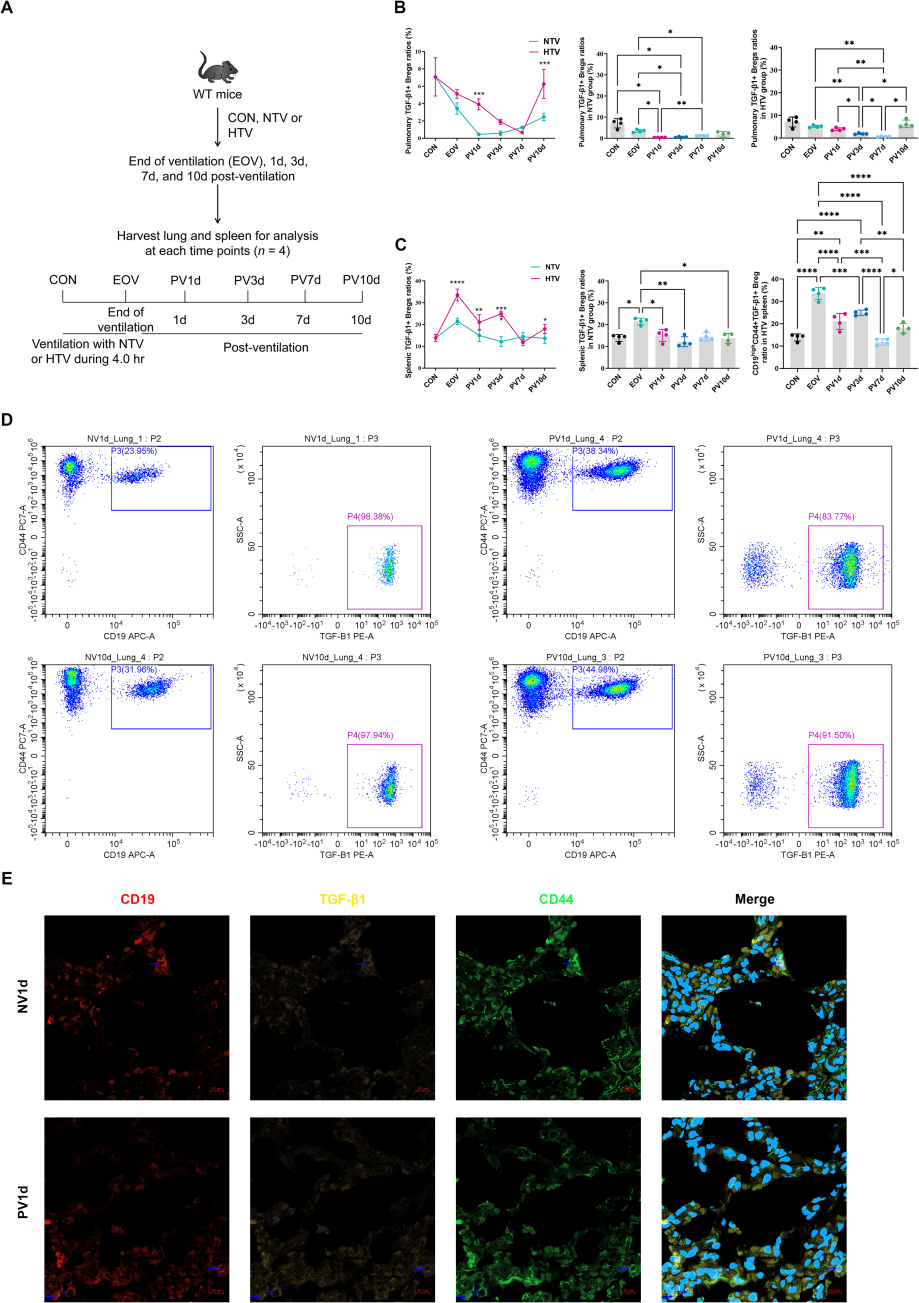
Temporal dynamics of TGF-β1-producing bregs in ventilator-induced lung injury resolution experimental timeline **(A)** and flow cytometric quantification of pulmonary **(B)** and splenic **(C)** TGF-β1+ Breg (pBreg) frequencies following high tidal volume (HTV) or normal tidal volume (NTV) ventilation. Representative flow profiles **(D)** and confocal microscopy **(E)** demonstrate enhanced pulmonary Breg infiltration in HTV mice at post-ventilation day 1 (scale: 10 μm). Data represent mean ± SEM (*n* = 4 mice/group). *P<0.05, **P<0.01, ***P<0.001, ****P<0.0001 *vs*. NTV controls.

Longitudinal analysis identified a two-phase Breg expansion in lungs: an acute peak at PV1d; 7.83-fold *vs*. NTV controls: *P*<0.001) followed by secondary resurgence at PV10d (**Figures 1B, D**). Conversely, splenic Breg peaked at end-of ventilation (EOV) before declining (**Figures 1C, D**). Confocal microscopy confirmed enhanced Breg infiltration in HTV-PV1d lungs (**Figure 1E**), corroborated by flow cytometry (**Supplementary Figure S2**).

### 3.2 Time-resolved pathological progression of VILI

**Figure 2:**
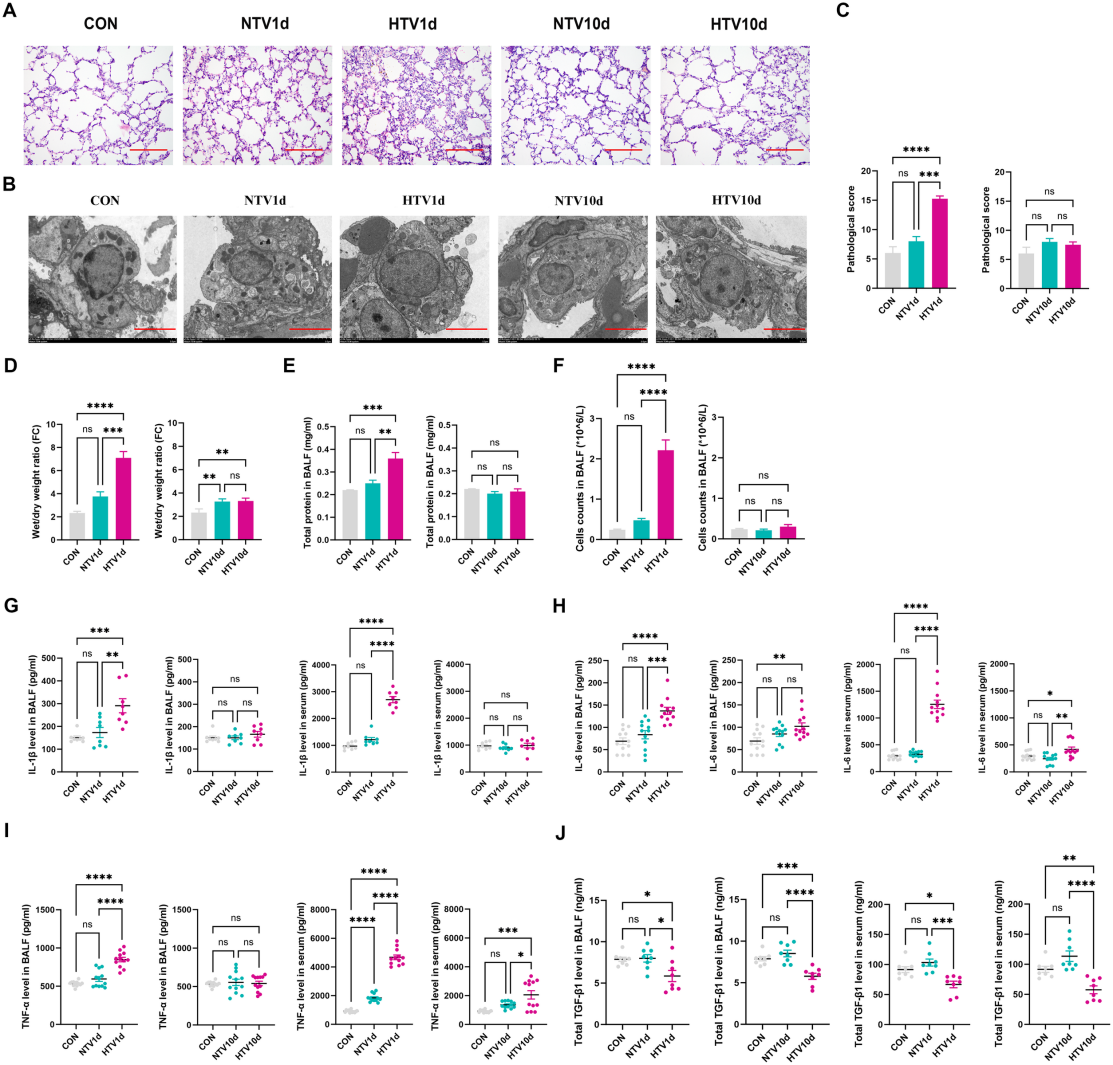
Biphasic pathophysiological progression of ventilator-induced lung injury. **(A)** Histopathological assessment shows alveolar hemorrhage and neutrophil infiltration in H&E-stained sections (scale bar: 100 μm). **(B)** Ultrastructural alveolar epithelial cell damage by transmission electron microscopy (scale bar: 5 μm). **(B–J)** Quantitative metrics during injury resolution include histopathology scores, lung wet/dry weight ratios, bronchoalveolar lavage fluid (BALF) protein and cell counts, and cytokine levels. Data are presented as mean ± SEM (n = 4 mice per group). *P < 0.05, **P < 0.01, ***P < 0.001, ***P < 0.0001 vs. CON group. NTV1d / NTV10d: NTV recovery at 1 and 10 days; HTV1d / HTV10d: HTV recovery at 1 and 10 days.

HTV-PV1d lungs exhibited hallmark histopathology—alveolar hemorrhage, neutrophil infiltration, and hyaline membranes—alongside ultrastructural damage to alveolar type II epithelial cells (mitochondrial swelling, lamellar body degeneration; **Figures 2A, B**). Quantitative metrics peaked at PV1d: lung wet/dry ratio increased 1.98-fold (*P* < 0.001 *vs*. sham), BALF protein rose 38% (*P* < 0.01), and proinflammatory cytokines (IL-1β, IL-6/, TNF-α) surged >4-fold (*P*<0.001, **Figures 2C–J**). Resolution occurred by PV10d despite persistent TGF-β1 elevation.

### 3.3 VILI-associated lymphocyte remodeling

**Figure 3:**
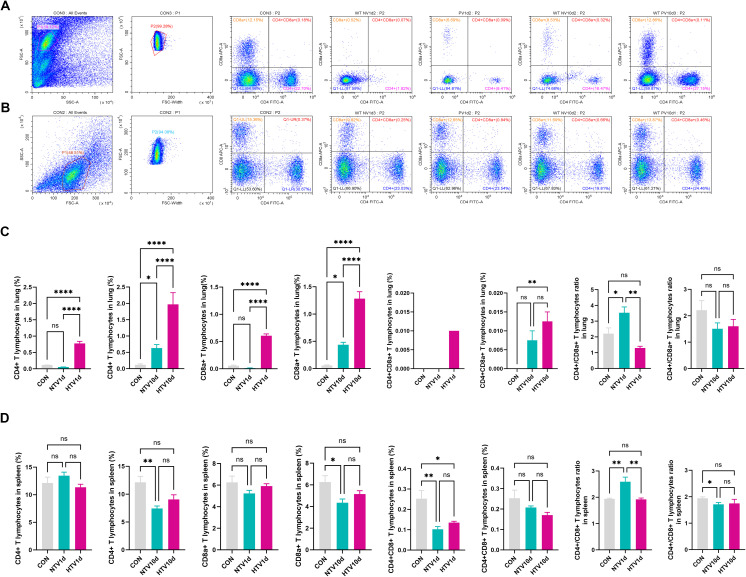
Ventilation-induced remodeling of pulmonary T cell subsets. Flow cytometric profiles showing pulmonary **(A)** and splenic **(B)** T cell immunophenotypes. Quantitative frequencies of CD4+, CD8a+, and double-positive (CD4+CD8a+) T cells in lung **(C)** and spleen **(D)** following mechanical ventilation. Data represent mean ± SEM (*n* = 4 mice/group). *P<0.05, **P<0.01, ***P<0.001, ****P<0.0001.

High-resolution immunophenotyping revealed dynamic T cell redistribution in VILI progression (**Figures 3A, B**). At PV1d, HTV-exposed lungs exhibited significant expansion of both CD4+ (4.3-fold vs CON, *P* < 0.0001) and CD8a+ T cells (6.1-fold vs NTV, *P* < 0.0001), whereas pulmonary CD4+/CD8a+ ratios were elevated in NTV1d versus CON and HTV1d groups (*P* < 0.05).

Longitudinal analysis demonstrated progressive accumulation of pulmonary CD4+CD8a+ double-positive T cells (DPTCs), with HTV10d showing 9.6-fold increase over CON (*P* < 0.01; **Figure 3C**). Conversely, splenic T cell subsets at PV1d showed no intergroup differences. Notably, both NTV1d and HTV1d cohorts displayed reduced splenic DPTC frequencies (≤0.4-fold vs CON, *P* < 0.01), while NTV1d maintained higher CD4+/CD8a+ ratios than CON and HTV1d (*P* < 0.01).

By PV10d, NTV mice exhibited significant splenic lymphopenia: CD4+ and CD8a+ T cell frequencies decreased 48% and 37% versus CON (*P* < 0.01), respectively, with concomitant reduction in CD4+/CD8a+ ratios (**Figure 3D**).

### 3.4 Enhanced therapeutic efficacy of TMNP

**Figure 4:**
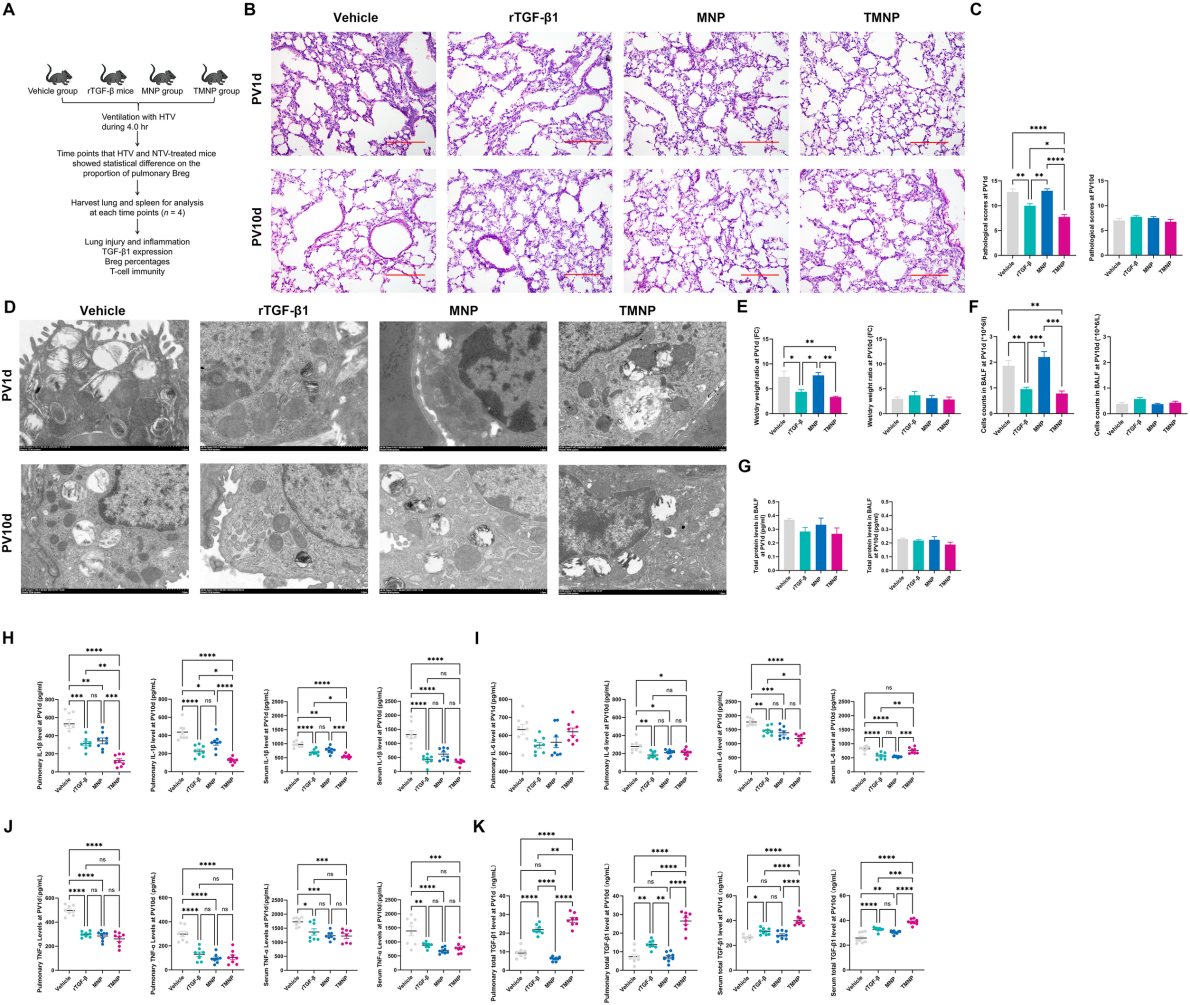
Therapeutic Efficacy of TGF-β1-loaded nanoparticles in Acute lung injury. Administration schema **(A)** and histological assessment (**B**, scale: 100 μm) showing TMNP-mediated protection. Quantitative outcomes include histopathology scores, ultrastructure preservation (TEM scale: 1 μm), edema reduction, and cytokine modulation **(C–K)**. Data represent mean ± SEM (*n* = 4 mice/group). *P<0.05, **P<0.01, ***P<0.001, ****P<0.0001.

TMNP demonstrated superior pharmacokinetics: sustained TGF-β1 release (35.8% EE) and colloidal stability (zeta potential: -26.7 mV; **Supplementary Figures S3F–H**). In HTV mice, TMNP administration significantly attenuated acute injury at PV1d (histopathology score reduced 22.5% *vs*. recombinant TGF-β1; *P*<0.01; **Figures 4B–F**) while maintaining 3.6-fold higher pulmonary TGF-β1 levels at PV10d (*P*<0.001*vs*. controls; **Figure 4K**), enabling prolonged immunomodulation.

### 3.5 TMNP reprograms lymphocyte crosstalk

**Figure 5:**
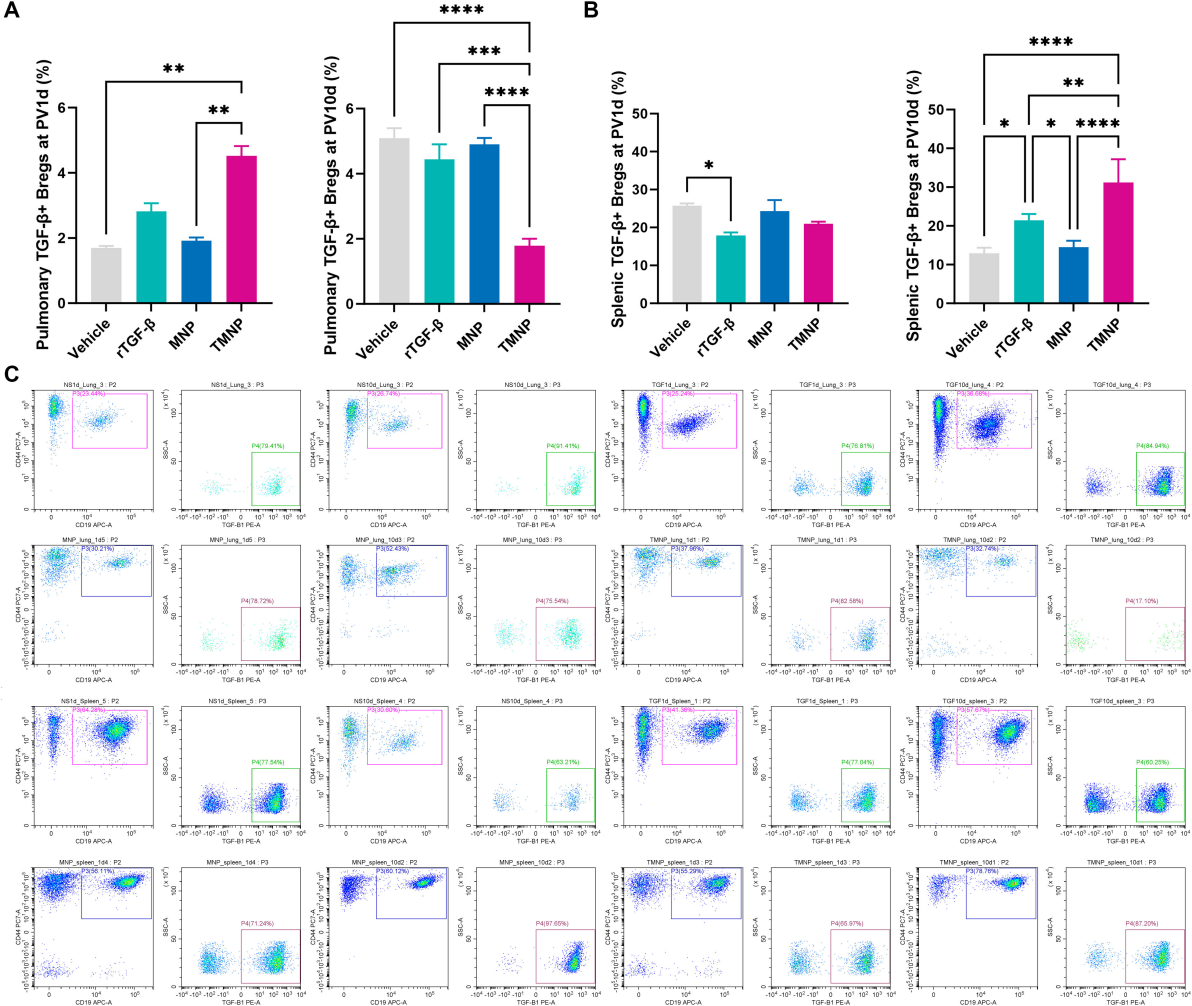
Spatiotemporal regulation of breg populations by nanoparticle therapy. Flow cytometric quantification demonstrating pulmonary **(A)** and splenic **(B)** Breg expansion following TMNP administration. Representative gating profiles illustrate subset dynamics **(C)**. Data represent mean ± SEM (*n* = 4 mice/group). *P<0.05, **P<0.01, ***P<0.001, ****P<0.0001.

**Figure 6:**
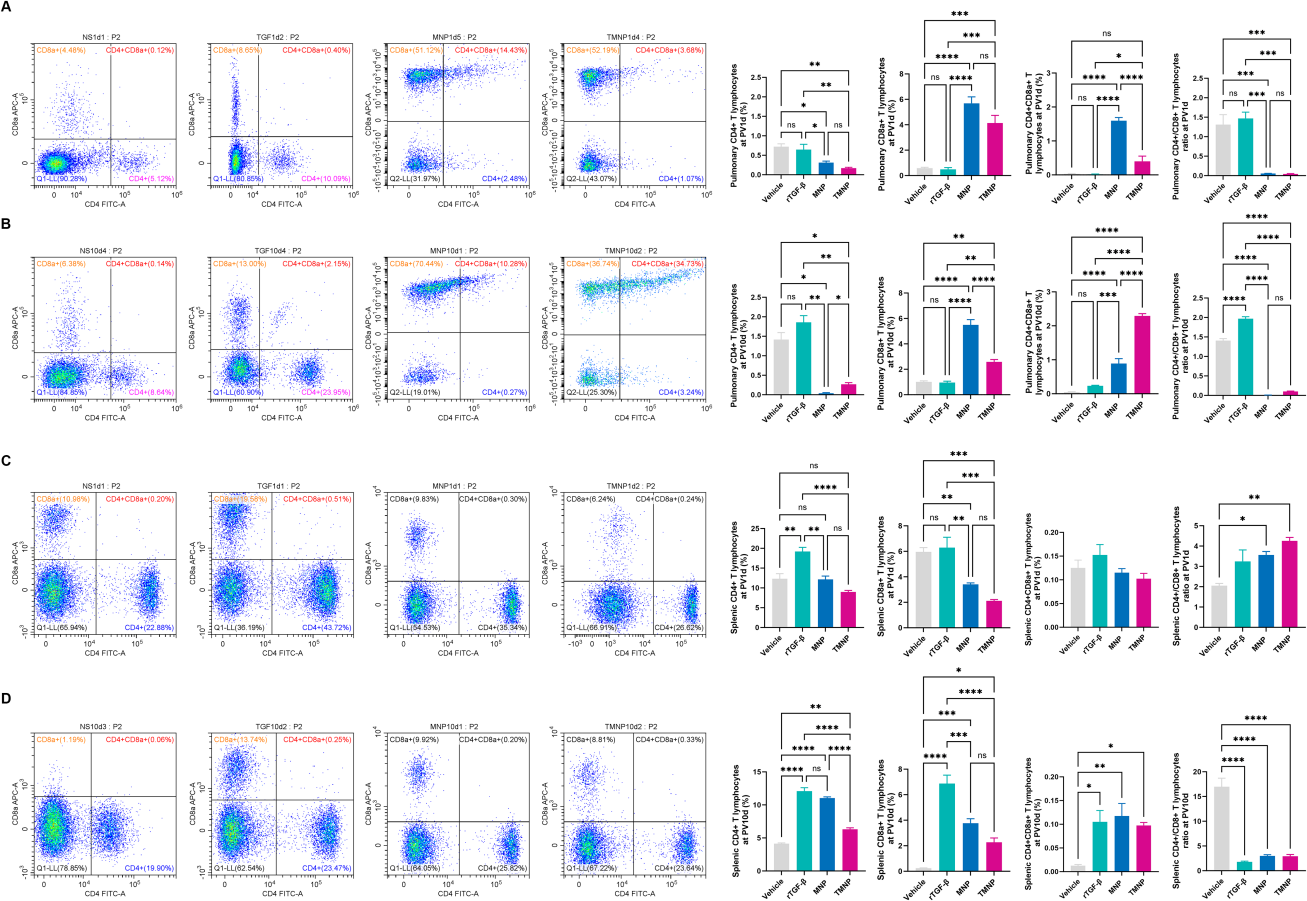
Nanoparticle-driven reprogramming of T cell Immunity. Temporal changes in pulmonary **(A, B)** and splenic **(C, D)** T cell subset frequencies following therapeutic intervention at post-ventilation days 1 and 10. Data represent mean ± SEM (*n* = 4 mice/group). *P<0.05, **P<0.01, ***P<0.001, ****P<0.0001.

Single-dose TMNP induced early pulmonary Breg expansion (PV1d: 2.66-fold *vs*. vehicle; *P*<0.001; **Figures 5A, C**) and late-phase splenic Breg polarization (PV10d: 59.7% increase *vs*. recombinant TGF-β1; *P*<0.01; **Figures 5B, C**). This coordinated response drove dynamic T cell remodeling: TMNP suppressed pulmonary CD4+ T cells (PV10d: 5.75 fold reduction *vs*. MNP; *P*<0.05) while expanding CD8a+ and double-positive T cells (DPTC; **Figures 6A–D**). Multivariate analysis revealed splenic Breg-CD4+CD8a+ T cell antagonism (r=0.559; **Supplementary Figure S4B**) and TGF-β1-mediated IL-6 suppression (r=-0.444; **Supplementary Figure S4A**).

## 4 Discussion

Our study establishes CD19^high^CD44^high^TGF-β1+ Bregs as spatiotemporal orchestrators of VILI resolution, with their therapeutic potential unlocked through biomimetic nanoparticle delivery. Building on previous work demonstrating IL-10+ Bregs involvement in chronic inflammation (20–22), we reveal TGF-β1+ Bregs exhibit phased activation: an acute pulmonary influx at PV1d to contain neutrophil extracellular traps (23, 24), followed by splenic priming atPV10d regulating CD4+CD8a+ T cell -mediated immunosuppression. This mirrors their dual role in cancer immunity—restraining early inflammation while permitting late-phase tolerance (25).

Nanotechnology-driven cytokine precision represents a transformative advance over conventional TGF-β1 therapy, which fails clinically due to pleiotropic effects and transient bioavailability (14). TMNP overcome these limitations through pH-responsive sustained release (>72 h *vs*. recombinant TGF-β1’s 6 h peak) (26) and Flotillin-2–mediated alveolar macrophage targeting (27). This aligns with emerging nanotherapeutic strategies for ARDS while demonstrating superior spatiotemporal control (28, 29).

We further identify CD4+CD8a+ T cells as TGF-β1+ Breg-regulated effectors in lung repair. Their expansion correlates inversely with splenic Breg activity (r=-0.72, *P* < 0.01) and parallels tumor-associated CD4+CD8a+ T cells that modulate CD8+ T cells via TGF-β1/PD-1 signaling (30), suggesting conserved immunosuppressive mechanisms across inflammatory contexts.

## 5 Clinical implications & limitations

While TMNP show compelling efficacy in acute inflammation, key questions require resolution: First, whether CD4+CD8a+ T cell expansion predispose to post-VILI fibrosis merits investigation using lineage-tracing models. Second, MMV coatings should be engineered to avoid tumor-promoting Breg phenotypes observed in cancer models (31). Crucially, human relevance must be established through humanized mouse systems—for example, NSG mice reconstituted with human hematopoietic stem cells could validate Breg dynamics across ventilation injury phases.

## 6 Concluding perspective

This work provides the first temporal mapping of TGF-β1+ Bregs in VILI, linking acute pulmonary infiltration to late splenic regulation of CD4+CD8a+ T cell interactions. Our MMV-based nanoformulation enables dual-phase immunomodulation: rapid injury containment and sustained homeostasis. By redefining CD4+CD8a+ T cell as key effectors in lung repair and demonstrating nanotechnology-enhanced cytokine delivery, we established a template for biomimetic therapeutics in ARDS management. Future studies should explore adoptive Breg transfer and cell-specific Tgfb1 deletion models to establish causal mechanisms.

## Data Availability

The original contributions presented in the study are included in the article/**Supplementary Material**. Further inquiries can be directed to the corresponding author.

## Supplementary material

Supplementary Figure 1 Molecular Signatures of TGF-β1+ Bregs in Ventilator-Induced Lung Injury. Flow gating gating strategy **(A)**, protein-protein interaction network **(B)**, and pathway enrichment analysis **(C, D)** characterizing regulatory B cell populations.

Supplementary Figure 2 Splenic Breg Dynamics during Injury Progression. Gating hierarchy **(A)** and quantitative frequencies **(B, C)** of splenic Breg subpopulations at end-of-ventilation and recovery timepoints (*n* = 4 mice/group).

Supplementary Figure 3 Biophysical Characterization of Macrophage-Mimetic Nanoparticles. Microvesicle biogenesis (**A, B**, scale: 20 μm), membrane protein validation **(C, D)**, electron microscopy (**E**, scale: 100 nm), colloidal stability **(F, G)**, sustained cytokine release profile **(H)**, and serum compatibility (I) of engineered nanotherapeutics.

Supplementary Figure 4 Systems-Level Correlation Networks in Lung Pathobiology. Pearson correlations matrices showing TGF-β1-IL-6 axis regulation **(A)**, Breg-T cell interactions **(B–D)**, lymphocyte subset interplay **(E–G)**, and biomarker relationships **(H–J)**. **P* < 0.05 after Bonferroni correction.
