# Correction: Spatiotemporal regulation of ventilator lung injury resolution by TGF-β1+ regulatory b cells via macrophage vesicle-nanotherapeutics

**DOI:** 10.3389/fimmu.2026.1886583

**Published:** 2026-05-28

**Authors:** Ren Jing, Xiaoting Liao, Jianlan Mo, Sheng He, Xianlong Xie, Zhaokun Hu, Linghui Pan

**Affiliations:** 1 Guangxi Clinical Research Center for Anesthesiology, Guangxi Medical University Cancer Hospital, Nanning, China; 2 Department of Breast and Thyroid Surgery, South China Hospital, Medical School, Shenzhen University, Shenzhen, China; 3 Department of Anesthesiology, Guangxi Medical University Cancer Hospital, Nanning, China; 4 Guangxi Engineering Research Center for Tissue & Organ Injury and Repair Medicine, Guangxi Medical University Cancer Hospital, Nanning, China; 5 Guangxi Key Laboratory for Basic Science and Prevention of Perioperative Organ Disfunction, Guangxi Medical University Cancer Hospital, Nanning, China; 6 Department of Anesthesiology, Guangxi Maternal and Child Health Hospital, Nanning, China; 7 The First Affiliated Hospital, Department of Anesthesiology, Hengyang Medical School, University of South China, Hengyang, Hunan, China; 8 Department of Intensive Care Unit, Guangxi Medical University Cancer Hospital, Nanning, China

**Keywords:** ventilation-induced lung injury, transforming growth factor-β1, regulatory B cells, immunoresolution, nanoparticles

There was a mistake in **Figure 2A** as published. The images for the NTV1d and NTV10d groups were misused. Upon re−examination of the original experimental data, we found that incorrect representative images had been selected during figure assembly. The images for the NTV1d and NTV10d groups have been replaced with correct images obtained from the same experimental conditions. The corrected **Figure 2A** appears below.

**Figure 2:**
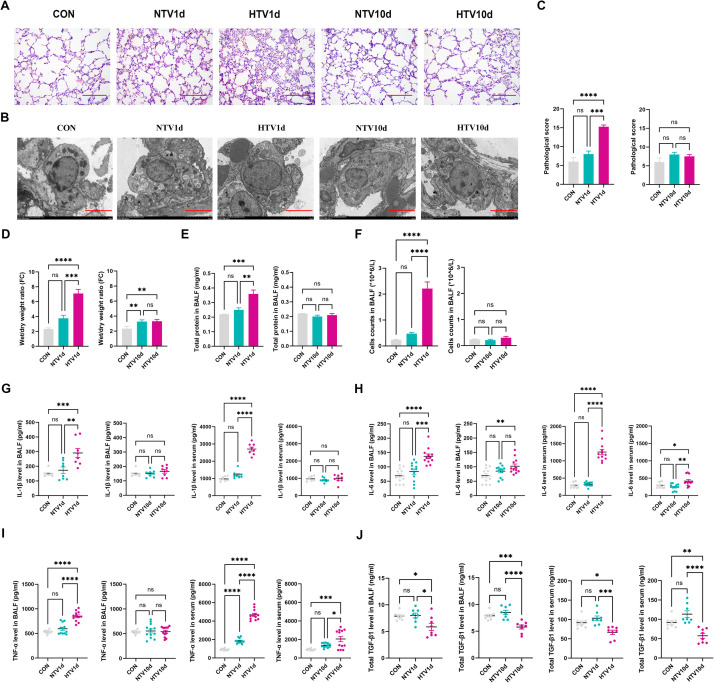
Biphasic pathophysiological progression of ventilator-induced lung injury. **(A)** Histopathological assessment shows alveolar hemorrhage and neutrophil infiltration in H&E-stained sections (scale bar: 100 μm). **(B)** Ultrastructural alveolar epithelial cell damage by transmission electron microscopy (scale bar: 5 μm). **(B–J)** Quantitative metrics during injury resolution include histopathology scores, lung wet/dry weight ratios, bronchoalveolar lavage fluid (BALF) protein and cell counts, and cytokine levels. Data are presented as mean ± SEM (n = 4 mice per group). *P < 0.05, **P < 0.01, ***P < 0.001, ***P < 0.0001 vs. CON group. NTV1d / NTV10d: NTV recovery at 1 and 10 days; HTV1d / HTV10d: HTV recovery at 1 and 10 days.

Also, there was a mistake in **Figure 4D**. The TEM image for the Vehicle_PV1d group was inadvertently taken from a previously published study by the same research group (Jing R, He S, Liao XT, et al. Transforming growth factor-β1 attenuates inflammation and lung injury with regulating immune function in ventilator-induced lung injury mice. Int. Immunopharmacol. 2023;114:109462). Specifically, that image was a high−magnification view of the same field as used in the earlier paper. To avoid duplicate publication, the TEM image for Vehicle_PV1d in **Figure 4D** has now been replaced with a new, representative high−magnification TEM image from the same experimental condition. The corrected **Figure 4D** appears below.

**Figure 4:**
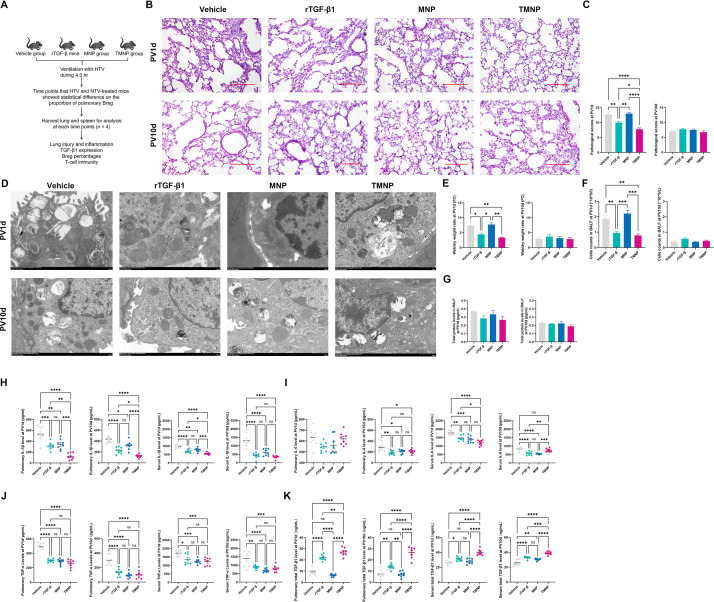
Therapeutic Efficacy of TGF-β1-loaded nanoparticles in Acute lung injury. Administration schema **(A)** and histological assessment [**(B)**, scale: 100 μm] showing TMNP-mediated protection. Quantitative outcomes include histopathology scores, ultrastructure preservation (TEM scale: 1 μm), edema reduction, and cytokine modulation **(C–K)**. Data represent mean ± SEM (n = 4 mice/group). *P<0.05, **P<0.01, ***P<0.001, ****P<0.0001.

The original version of this article has been updated.

