# Supplementary material for: Spatiotemporal regulation of ventilator lung injury resolution by TGF-β1+ regulatory B cells via macrophage vesicle-nanotherapeutics
Source: Front Immunol. 2025 Jul 10;16:1635178. doi: 10.3389/fimmu.2025.1635178 (PMC12286806; doi:10.3389/fimmu.2025.1635178)
Supplement: Supplementary Figure 1 — Molecular Signatures of TGF-β1+ Bregs in Ventilator-Induced Lung Injury. Flow gating gating strategy (A), protein-protein interaction network (B), and pathway enrichment analysis (C, D) characterizing regulatory B cell populations. [file DataSheet1.doc]

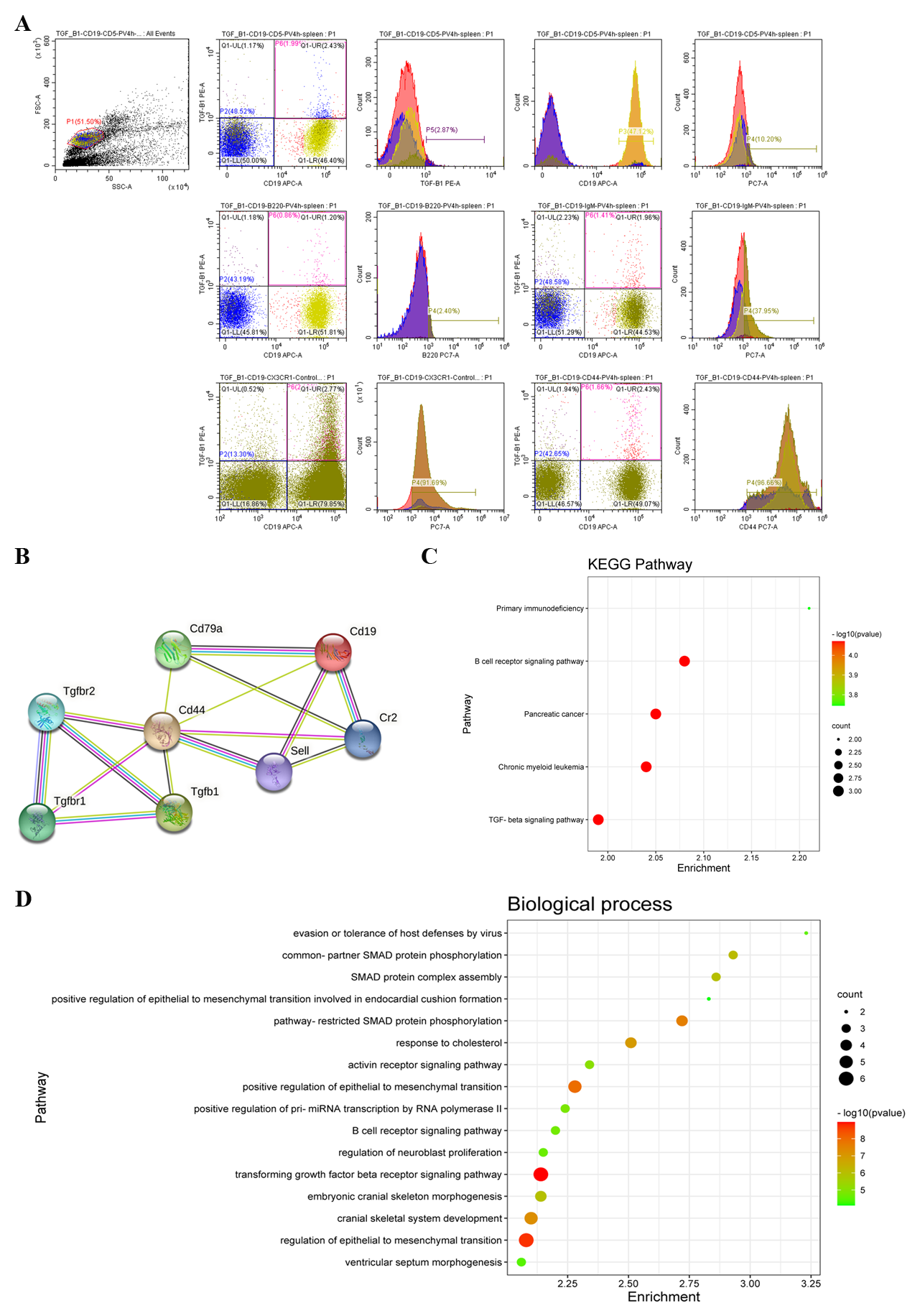
**Supplementary Figure S1. Molecular Signatures of TGF-β1+ Bregs in Ventilator-Induced Lung Injury**

Flow gating gating strategy (A), protein-protein interaction network (B), and pathway enrichment analysis (C-D) characterizing regulatory B cell populations.


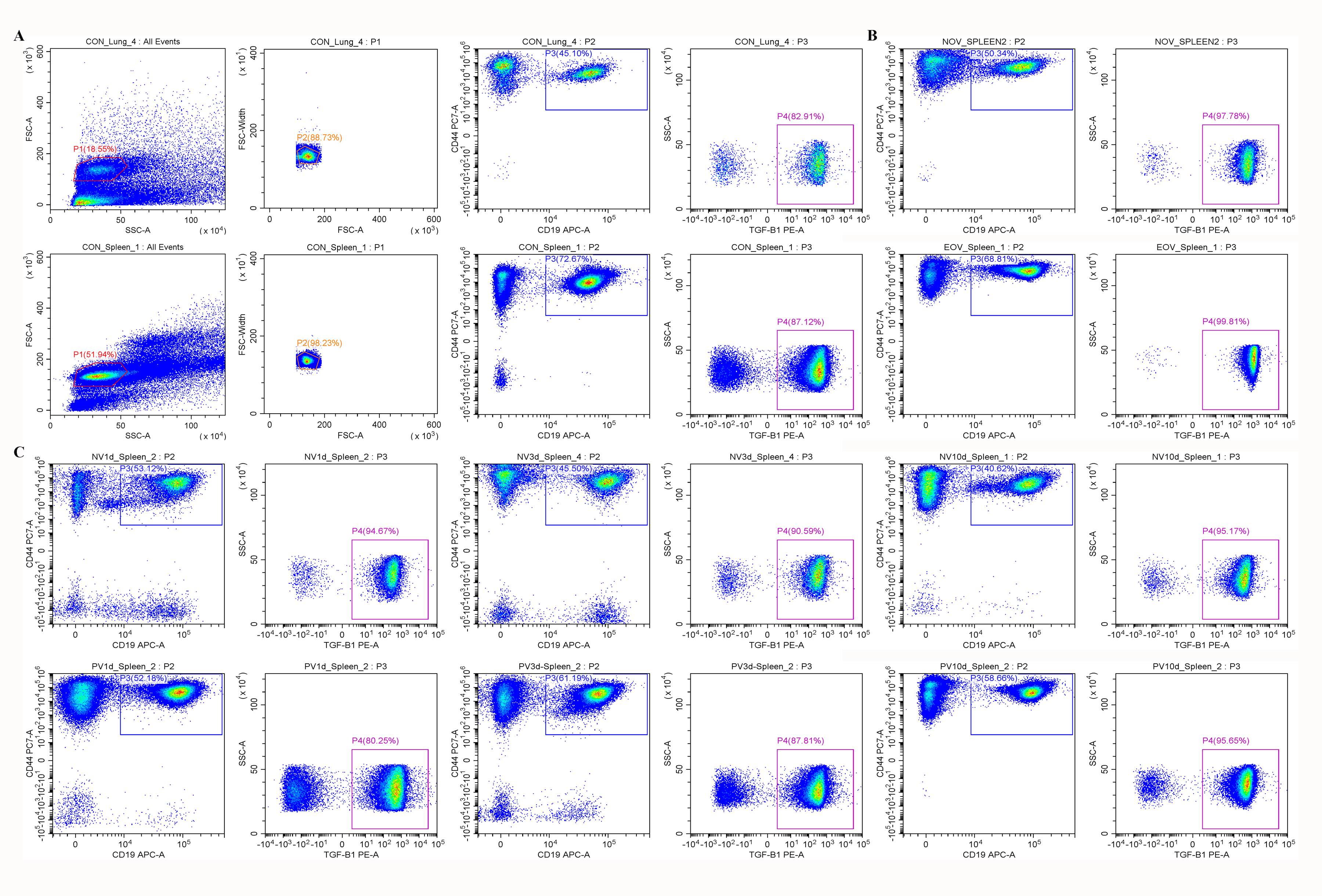
**Supplementary Figure S2. Splenic Breg Dynamics during Injury Progression**

Gating hierarchy (A) and quantitative frequencies (B-C) of splenic Breg subpopulations at end-of-ventilation and recovery timepoints (*n* = 4 mice/group).


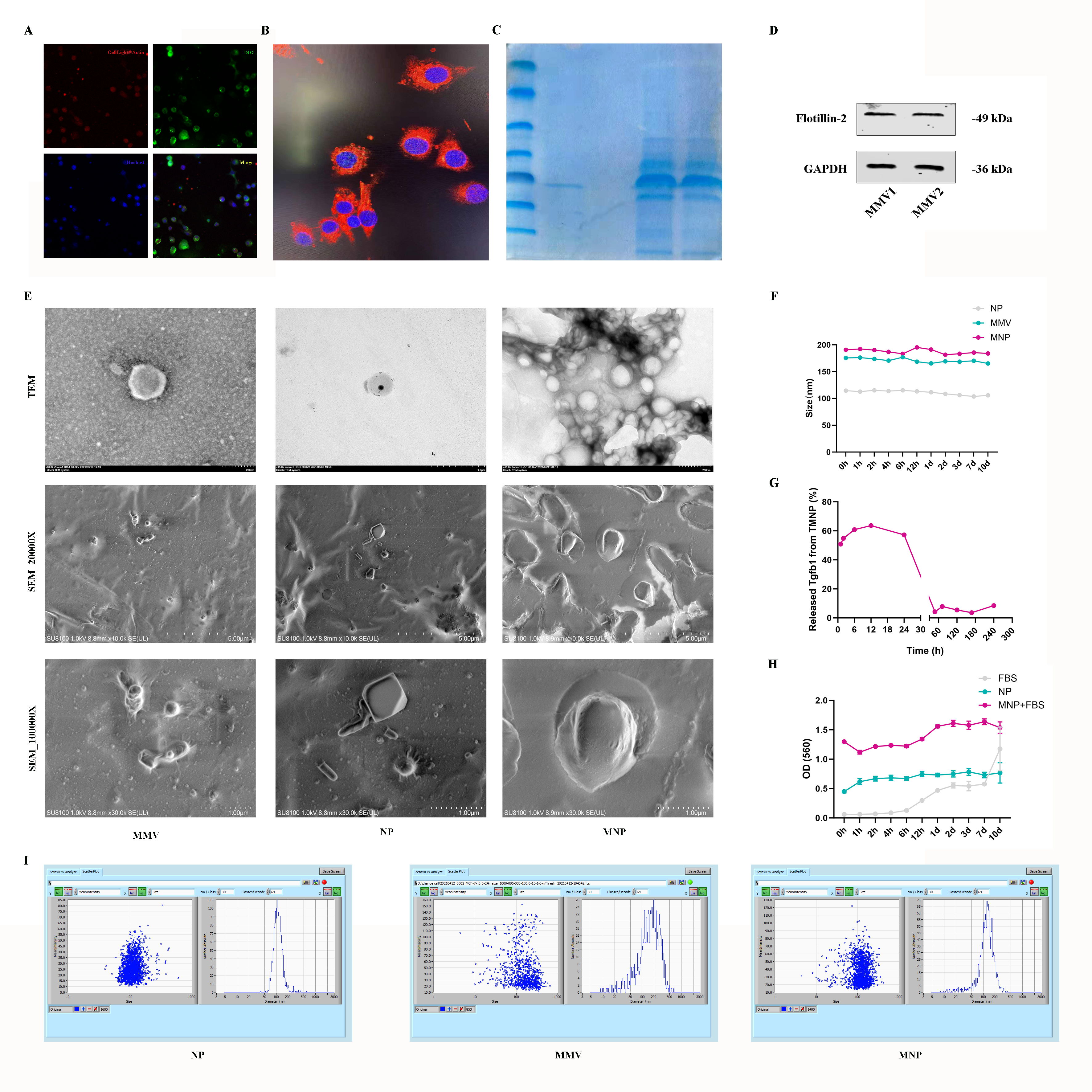
**Supplementary Figure S3. Biophysical Characterization of Macrophage-Mimetic Nanoparticles**

Microvesicle biogenesis (A-B, scale: 20 μm), membrane protein validation (C-D), electron microscopy (E, scale: 100 nm), colloidal stability (F-G), sustained cytokine release profile (H), and serum compatibility (I) of engineered nanotherapeutics.


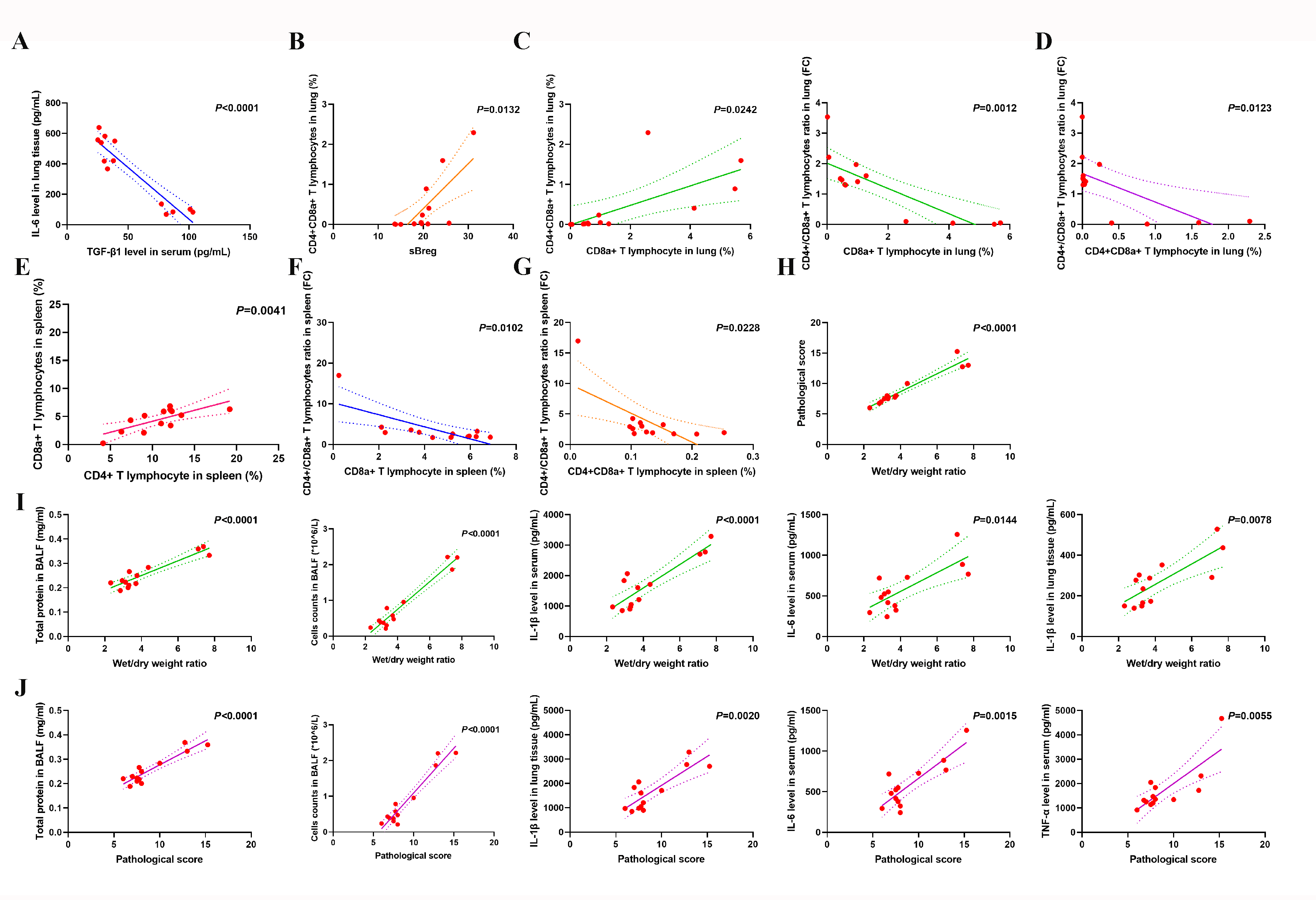
**Supplementary Figure S4. Systems-Level Correlation Networks in Lung Pathobiology**

Pearson correlations matrices showing TGF-β1-IL-6 axis regulation (A), Breg-T cell interactions (B-D), lymphocyte subset interplay (E-G), and biomarker relationships (H-J). **P* < 0.05 after Bonferroni correction.
